# Phase Stability of Nanocrystalline Grains of Rare-Earth Oxides (Sm_2_O_3_ and Eu_2_O_3_) Confined in Magnesia (MgO) Matrix

**DOI:** 10.3390/ma13092201

**Published:** 2020-05-11

**Authors:** Chen Barad, Giora Kimmel, Hagay Hayun, Dror Shamir, Kachal Hirshberg, Yaniv Gelbstein

**Affiliations:** 1 The Unit of Energy Engineering, Ben-Gurion University of the Negev, Beer-Sheva 84105, Israel; chenhu@post.bgu.ac.il; 2 NRCN, P.O. Box 9001, Beer-Sheva 84190, Israel; drorshamir@gmail.com; 3 Institutes for Applied Research, Ben-Gurion University of the Negev, Beer-Sheva 84105, Israel; gyorakimmel@gmail.com; 4 Department of Materials Engineering, Ben-Gurion University of the Negev, Beer-Sheva 84105, Israel; hagayha@post.bgu.ac.il (H.H.); kachal9227@gmail.com (K.H.)

**Keywords:** oxide materials, sol-gel, phase transformations, X-ray diffraction, scanning electron microscopy (SEM)

## Abstract

Rare-earth (RE) oxides are important in myriad fields, including metallurgy, catalysis, and ceramics. However, the phase diagram of RE oxides in the nanoscale might differ from the phase diagrams for bulk, thus attracting attention nowadays. We suggest that grain size in the nanoscale also determines the obtained crystallographic phase along with temperature and pressure. For this purpose, nanoparticles of Sm_2_O_3_ and Eu_2_O_3_ were mixed in an inert MgO matrix via the sol-gel method. This preparation method allowed better isolation of the oxide particles, thus hindering the grain growth process associated with increasing the temperature. The mixed oxides were compared to pure oxides, which were heat-treated using two methods: gradual heating versus direct heating to the phase transition temperature. The cubic phase in pure oxides was preserved to a higher extent in the gradual heating treatment compared to the direct heating treatment. Additionally, in MgO, even a higher extent of the cubic phase was preserved at higher temperatures compared to the pure oxide, which transformed into the monoclinic phase at the same temperature in accordance with the phase diagram for bulk. This indicates that the cubic phase is the equilibrium phase for nanosized particles and is determined also by size.

## 1. Introduction

Rare-earth (RE) oxides have gained popularity over the years owing to their versatile applications, such as the nuclear field (Sm_2_O_3_ is used as an absorber in control rods), solid oxide fuel cells (Sm_2_O_3_-doped ceria electrolyte was found to have a high ionic conductivity), phosphor materials (Eu_2_O_3_ is used as red or blue phosphor), catalysis, laser, optical, and more [1,2,3].

The crystallographic structure influences the physical and chemical properties of RE oxides and thus determines their functionality. Hence, achieving a better understanding of phase stability behavior has been a continuous research aim, but mainly regarding the influence of pressure and temperature rather than that of crystal size [4,5,6,7]. 

All the rare-earth elements form a sesquioxide of RE_2_O_3_. Regarding the polymorphism of rare-earth oxides, five different crystallographic phases are known: at temperatures lower than about 2273 K, three types marked as A (hexagonal *P32/m*), B (monoclinic *C2/m*), and C (cubic *Ia3*) are usually observed; and for temperatures higher than 2273 K, the crystallographic phases marked as H (hexagonal) and X (cubic) are formed [8]. When increasing the temperature, the order of transition is generalized as C 🡢 B 🡢 A, even though not every oxide will show all phases. This common transition is characteristic of the intermediate elements of the group [4,9,10,11,12].

The cubic polymorph of Eu_2_O_3_, for instance, transforms to the monoclinic polymorph at approximately 1473 K, and then to the hexagonal polymorph at approximately 2067 K. By additional heating, the hexagonal polymorph transforms to the H polymorph at approximately 2167 K, and lastly to the X form at approximately 2553 K just before the melting point at 3133 K is reached. Regarding Sm_2_O_3_, both the cubic and the monoclinic polymorphs exist: the cubic phase is stable at room temperatures up to approximately 1173 K, at which the monoclinic polymorph is formed [4,13,14].

Today, increasingly more attention is being directed towards the preparation and research of ultrafine particles of rare-earth oxides in the nano-range with the aim of lowering the sintering temperature, improving mechanical properties of the final product, and enhancing super-elastic properties for net-shape-forming purposes [15,16].

Most crystallographic phase diagrams in the literature are for bulk materials, and only a few present or discuss the phase diagrams of the material in its nanometric range (approximately 5–100 nm) [17,18,19,20]. The differences between nano and bulk materials are not always fully understood or even known. However, in the era of nanomaterials, those differences are important to comprehend, since they affect the final product properties.

Even though the effect of crystallite size on the crystallographic structure of nanoparticles is a concept that is gaining more recognition nowadays [17,20,21], this effect is not fully understood and tends to remain marginal. Understanding the role of crystallite size on structure might change the conservative agenda towards phase diagrams when dealing with nanoparticles.

This study investigates the crystallographic phases in some rare-earth oxides from the middle range of ionic radius in the group (Sm_2_O_3_ and Eu_2_O_3_), both as pure oxides and as embedded nanoparticles in an inert MgO matrix. In order to better heighten the grain size effect (as grains tend to grow with increasing temperature), the crystallographic phases were investigated in systems where the desired oxide was embedded in a MgO matrix in which the grain growth process tends to be deliberately hindered [20,21,22].

The MgO-RE_2_O_3_ systems and the pure oxides were produced via the sol-gel method. The sol-gel method was chosen because it is known as a technique for producing nano and homogenous powders in controllable concentrations in room conditions. The obtained xerogels were gradually heated to the desired temperature and calcined for 2 h in air. The pure oxides were also heat-treated identically and were used as control samples. In addition, the pure oxides were also heat-treated in a different manner including direct heating of the sample at the final and desired phase transition temperature for 2 h by placing the sample immediately in the furnace at the required temperature. The two different heating procedures for the pure oxides were performed in order to also investigate the influence of thermal treatment parameters on the obtained crystallographic phases in pure oxides. The crystal structure and the microstructure were characterized by X-ray diffraction (XRD) and high-resolution scanning electron microscopy (HR-SEM). 

## 2. Materials and Methods

RE_2_O_3_ (RE = Sm and Eu) was confined in a magnesium oxide (MgO) matrix via the sol-gel method, which resulted in a variety of compositions: 5, 10, 20, and 40 vol % of RE_2_O_3_ in MgO. As control, pure Sm_2_O_3_ and Eu_2_O_3_ were also synthesized via the sol-gel method. The powder batches were synthesized by using magnesium nitrate hexahydrate [Mg(NO_3_)_2_·6H_2_O] (purity ~98%), samarium nitrate hexahydrate [Sm(NO_3_)_3_·6H_2_O] (purity ~99.95), and europium nitrate hydrate [Eu(NO_3_)_3_·5H_2_O] (purity ~99.9%). Precursors (hygroscopic salts) were vacuum dried before adding stoichiometric amounts to 100 mL of deionized water with 18.3 MΩ·cm resistance. The continuous stirred reactor was placed in an ice bath in order to induce precipitation of more soluble magnesium hydroxide (Mg(OH)_2_). After a hydrolysis of precursors and obtaining a homogeneous solution, an ammonium hydroxide solution (2 N) was dripped slowly into the mixture, which turned more milky and viscous with each drop. Finally the gel point was observed at a pH value of 10, and the dripping stopped. The gel’s precipitates were dried in an air furnace at 343 K for 4 days. The powder was ground using an agate mortar and divided into different sample batches, which were gradually heated (5 K/min) in air to the desired calcination temperature (in the range of 873–1473 K) using alumina (Al_2_O_3_) crucibles, and then calcined for 2 h in order to demonstrate the transformation of crystallographic phase transitions. Samples of pure oxides were also immediately placed in the furnace at the phase transition temperature and calcined for 2 h in order to compare this process with the previously mentioned thermal treatment.

X-ray diffraction (XRD; DMAX 2100 powder diffractometer, Rigaku, Japan) studies were performed using powder diffraction with Cu Kα_1_ radiation. The XRD patterns were analyzed using the Powder-Cell program [23], and Rietveld refinement was also carried out using the FullProf/ WinPLOTR software (T. Roisnel\J. Rodriguez-Carvajal, CDIFX UMR6226 Rennes\ILL Grenoble, France) [24,25]. The morphology and chemical composition was investigated using a high-resolution scanning electron microscope (HR-SEM; JSM-7400, JEOL, Tokyo, Japan) with back scattered electrons detector (BSE) contrast and energy dispersive spectroscopy (EDS; JSM-7400, JEOL, Tokyo, Japan). The accelerating voltage and working distance were 10 kV and 6.1 mm, respectively. The samples were fixed on a graphite tape and coated with a thin layer of carbon in order to increase the electrical conductivity. The composition analysis of the sol-gel powder was also investigated by inductively coupled plasma optic emission spectroscopy (ICP-OES; Spectro Arcos, Kleve, Germany). The crystal size (below 60 nm) was evaluated based on XRD analysis using the Williamson-Hall approach and by HR-SEM using imageJ (image processing software; National Institutes of Health, Bethesda, MD, USA) in the higher nano-range (above 60 nm).

## 3. Results

ICP-OES results confirmed that following the sol-gel synthesis, the powder composition was consistent with the theoretical design (maximal deviation of ~5%). The results are shown in Table 1.

**Figure 1:**
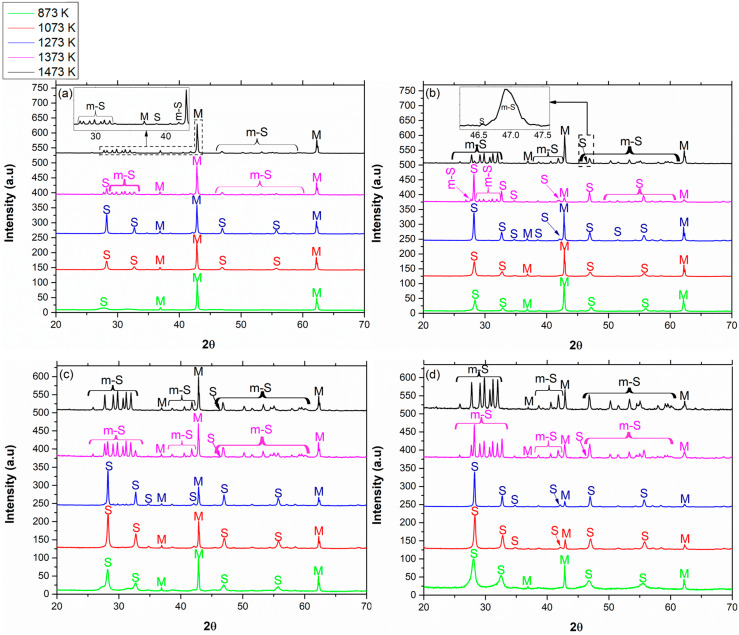
XRD patterns of the binary system of Sm_2_O_3_ in MgO in different concentrations: (**a**) 5 vol %, (**b**) 10 vol %, (**c**) 20 vol %, and (**d**) 40 vol % calcined at different calcination temperatures for 2 h.

The XRD patterns of MgO with different concentrations of Sm_2_O_3_ calcined at different calcination temperatures are shown in Figure 1. All samples were gradually heated to the final temperature and were calcined for 2 h. Peaks originated from Sm_2_O_3_ are marked by “S”, and those originated from MgO are marked by “M”. In cases where the monoclinic phase of the rare-earth oxide was apparent, it was marked by “m-S”.

**Figure 2:**
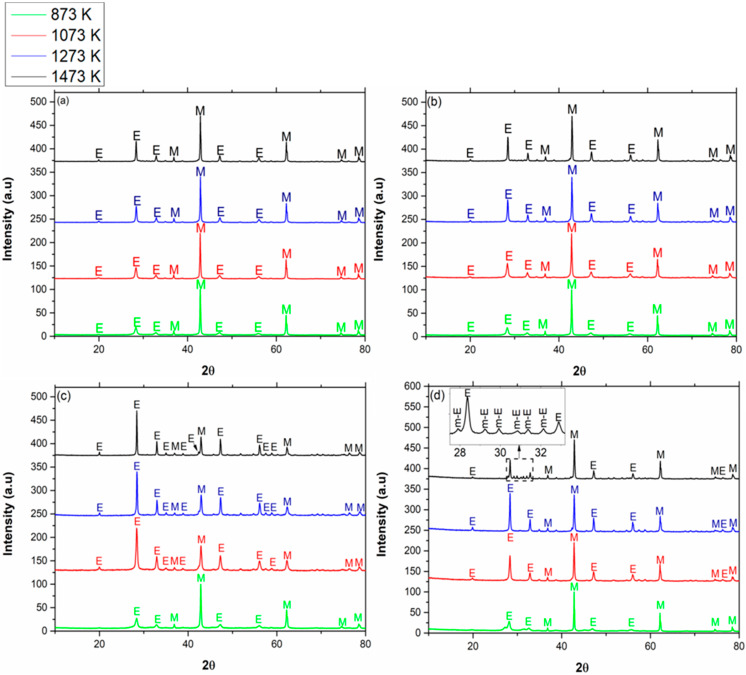
XRD patterns of the binary system of Eu_2_O_3_ in MgO in different concentrations: (**a**) 5 vol %, (**b**) 10 vol %, (**c**) 20 vol %, and (**d**) 40 vol %, calcined at different calcination temperatures for 2 h.

**Figure 3:**
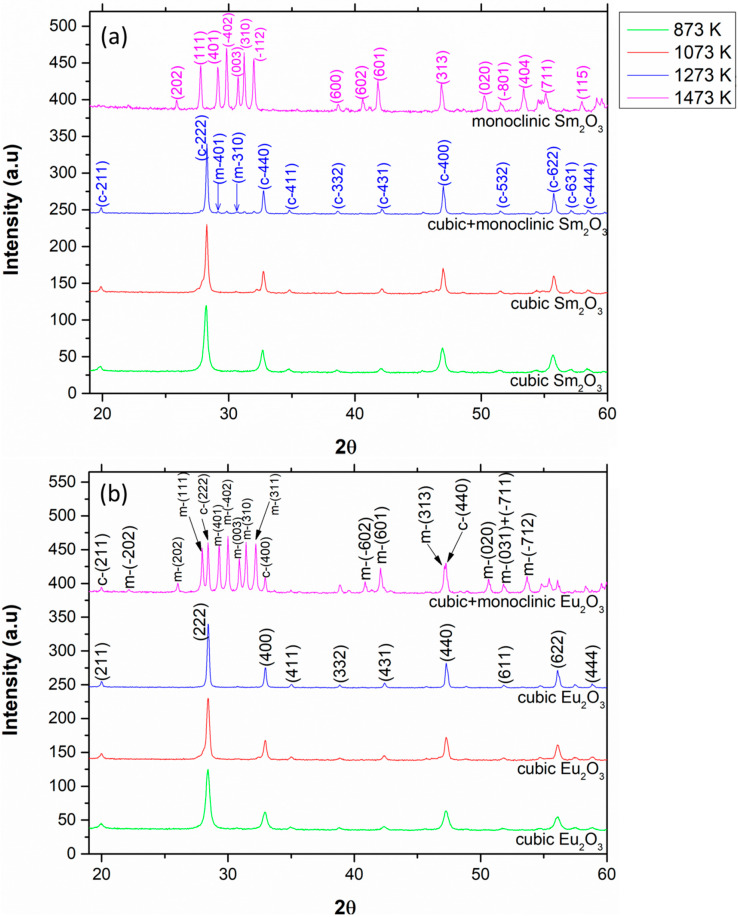
XRD patterns of pure (**a**) Sm_2_O_3_ and (**b**) Eu_2_O_3_ heated gradually and then calcined at different calcination temperatures for 2 h.

In all compositions, for calcination temperatures of 873-1273 K, the Sm_2_O_3_ was in the cubic phase and began the c 🡢 m transition at calcination temperatures of 1373–1473 K (mixture of cubic and monoclinic). A sole monoclinic phase was observed at 1473 K (2 h) for 40 vol % Sm_2_O_3_ in MgO only. On the other hand, according to Figure 3a, pure Sm_2_O_3_ already started the c 🡢 m transition at 1273 K (in accordance with Roth and Schneider [11]), and this transition was completed at 1473 K. Hence, the transition from the cubic to the monoclinic phase was delayed for all tested concentrations of Sm_2_O_3_ in MgO in comparison with pure Sm_2_O_3_. The crystallite size was calculated via the Williamson-Hall plot:

(1) β·cos(θ)=k·λL+4·ε·sin(θ)

where *β* is the broadening of the diffraction line measured at half of the maximum intensity, *λ* is the wavelength (Cu-κα), *θ* is the Bragg angle for a given diffraction, and *k* is a constant, which is in general equals 0.94 for powders. The instrumental broadening effect was estimated by subtracting the full width at half-maximum of a standard sample (LaB_6_) from *β* of the respective Bragg peaks. L represents the crystallite size, and *Ԑ* represents the lattice strain. The characteristic Williamson-Hall plot corresponds to the graph of *β*·cos(*θ*) versus sin(*θ*). After data collection, a linear regression should give a linear fit. The crystallite size was extracted from the y-intercept of the (k·λL) fit. Regarding the grain size, grains of cubic Sm_2_O_3_ in MgO were smaller (broader peaks) in comparison with pure Sm_2_O_3_ (sharper peaks) for the same calcination temperature (for example: for 5–40 vol % Sm_2_O_3_ in MgO calcined at 1273 K, the average cubic grain size was in the range of 30–80 nm, respectively, while for pure Sm_2_O_3_, the average grain size was greater than 100 nm). Table 2 summarizes the experimental results for Sm_2_O_3_:

The XRD patterns of MgO with different concentrations of Eu_2_O_3_ calcined at different calcination temperatures are shown in Figure 2. All samples were gradually heated to the final temperature and were calcined for 2 h. Peaks originated from Eu_2_O_3_ are marked by “E”, and those originated from MgO are marked by “M”. In cases where the monoclinic phase of the rare-earth oxide was apparent, it was marked by “m-E”.

In compositions of 5, 10, and 20 vol % Eu_2_O_3_ in MgO, the Eu_2_O_3_ remained cubic at each calcination temperature in the range of 873–1473 K. On the other hand, according to Figure 3b, pure Eu_2_O_3_ was mostly monoclinic at 1473 K (approximately 83%). Hence, the transition from the cubic to the monoclinic phase was delayed in the MgO-Eu_2_O_3_ systems containing 5, 10, and 20 vol % Eu_2_O_3_ in MgO. Regarding grain size, grains of cubic Eu_2_O_3_ in MgO were smaller (broader peaks) in comparison with pure Eu_2_O_3_ (sharper peaks) for the same calcination temperature (for example: for 5 vol % Eu_2_O_3_ in MgO calcined at 1473 K, the grain size was approximately 30 nm, while for pure Eu_2_O_3_, the average grain size was greater than 100 nm). Table 3 summarizes the experimental results for Eu_2_O_3_.

According to Table 2 and Table 3, in general, for a given concentration of the RE_2_O_3_ in MgO, crystal size increases when raising the calcination temperature. In addition, for a given calcination temperature, crystal size increases when the concentration of the RE_2_O_3_ in MgO increases. Thus, crystal growth is more suppressed when a smaller amount of the RE_2_O_3_ is embedded in the MgO matrix. Moreover, the monoclinic structure was obtained when large enough crystals were formed, while smaller crystals remained cubic. Thus, the presence of a mixture of the two phases (cubic and monoclinic) is due to non-uniformity in crystal size.

In all samples, the lattice parameter of MgO was normal and nearly a constant (4.212 ± 0.002 Å). In general, the RE_2_O_3_-MgO systems have a lower lattice parameter compared with the pure oxides, and for a given concentration, the unit cell parameter decreased as temperature increased. The MgO matrix exerted hydrostatic pressures on the embedded oxide during cooling down caused by the higher thermal expansion coefficient of the MgO matrix [26,27].

In the monoclinic structure, the mean distance between ions is smaller, and the bond energy is minimal. On the other hand, the cubic structure is characterized by larger distances between the ions, but this structure is isometric and therefore has higher entropy. In systems with nanosized particles (high surface area to volume ratio), the effect of surface area becomes significant and affects the Gibbs energy of each phase [19,28,29,30]; therefore, systems of nanoparticles show different behaviour in comparison with bulk materials. Here, in powder with smaller crystallites, the cubic phase (low surface energy phase) is favoured. In other words, in nanometric crystals, the surface energy plays a more dominant role, and there is a preference for the cubic structure rather than the monoclinic. Above a certain size, the surface energy is no longer a dominant factor, and the cubic phase transforms into the monoclinic phase. A similar effect was shown regrading other oxides such as plasma sprayed films of Y_2_O_3_ [31], and tetragonal ZrO_2_ and gamma Al_2_O_3_ synthesized from aqueous solutions [19,20].

The XRD patterns of pure Sm_2_O_3_ and Eu_2_O_3_ calcined at different calcination temperatures are shown in Figure 3.

**Figure 4:**
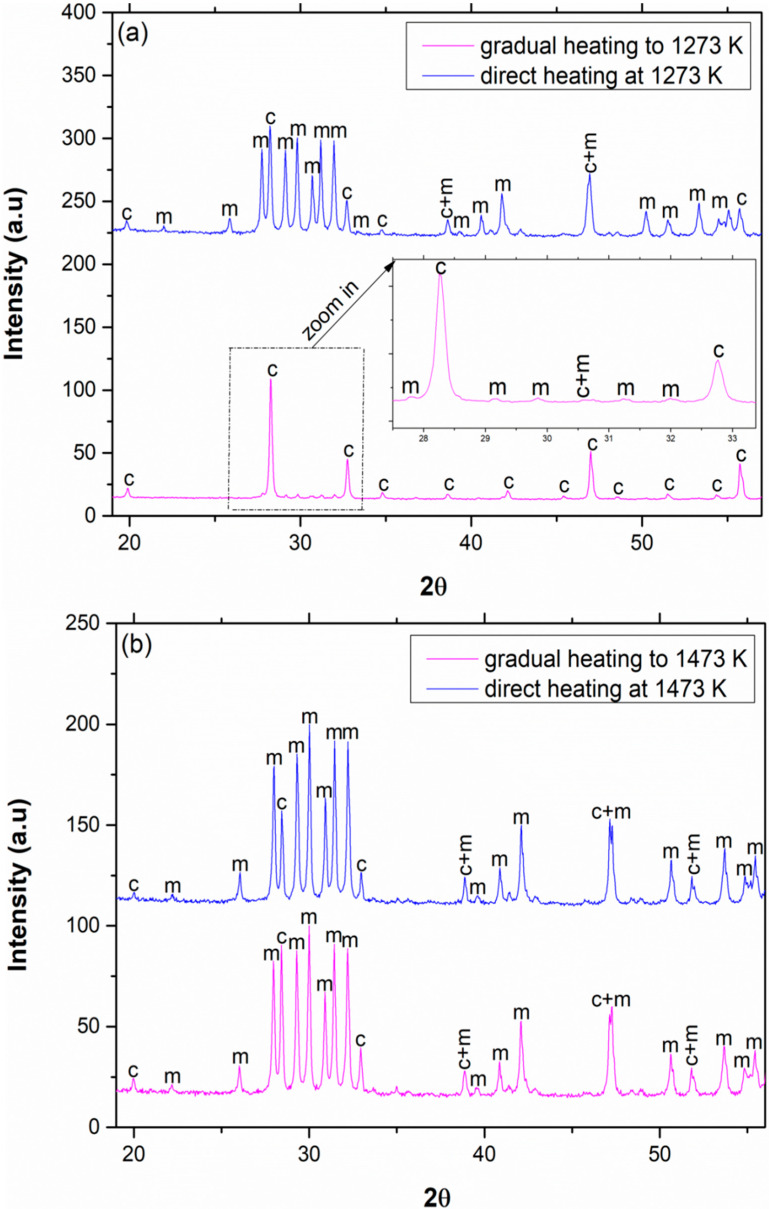
XRD patterns of (**a**) pure Sm_2_O_3_ gradually and directly calcined at 1273 K for 2 h and (**b**) pure Eu_2_O_3_ gradually and directly calcined at 1473 K for 2 h.

Pure Sm_2_O_3_ started the c 🡢 m transformation at 1273 K (9% monoclinic) and completed the transition at 1473 K, while pure Eu_2_O_3_ started the transformation at 1473 K (83% monoclinic). The transition temperatures for both oxides are in accordance with the traditional phase diagram [11]. However, the low fraction of the monoclinic phase at the transition temperatures (especially for Sm_2_O_3_) indicates a possible suppression during the grain growth process that might be associated with the thermal treatment procedure. A comparison between the two different thermal treatment processes regarding pure Sm_2_O_3_ and Eu_2_O_3_ is shown in Figure 4. 

The phase composition of pure Sm_2_O_3_ was 9% monoclinic and 91% cubic when heated gradually to 1273 K and then calcined for 2 h at 1273 K. However, when pure Sm_2_O_3_ was placed directly in the furnace at 1273 K for 2 h, the monoclinic content increased to approximately 73%. Hence, the heat treatment parameters influence the final crystallographic phase composition. Regarding pure Eu_2_O_3_, the content of the monoclinic phase slightly increased from 80% to approximately 90% when heated directly for 2 h at 1473 K. This proves that the transition is size-dependent. In addition, according to Roth and Schneider [11], the progression of the c 🡢 m transformation is governed also by crystallinity.

**Figure 5:**
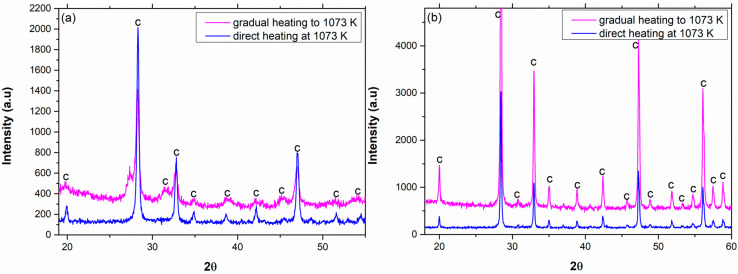
XRD patterns of (**a**) pure Sm_2_O_3_ gradually (in red) and directly (in blue) calcined at 1073 K for 2 h and (**b**) pure Eu_2_O_3_ gradually (in red) and directly (in blue) calcined at 1073 K for 2 h.

A similar comparison was also executed regarding pure Sm_2_O_3_ and Eu_2_O_3_ calcined at temperatures below the phase transition temperature, as shown in Figure 5.

**Figure 6:**
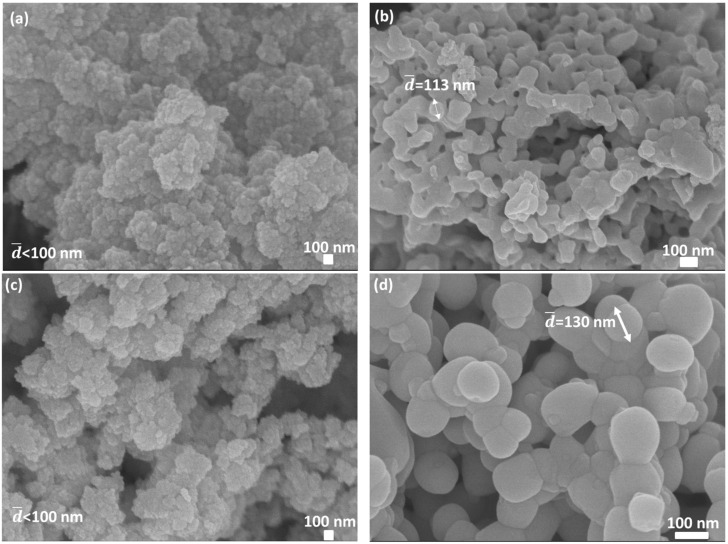
High-resolution scanning electron microscopy (HR-SEM) images of (**a**) Sm_2_O_3_ calcined at 1073 K for 2 h (gradual heating), (**b**) Sm_2_O_3_ calcined at 1073 K for 2 h (direct heating), (**c**) Eu_2_O_3_ calcined at 1073 K (gradual heating), and (**d**) Eu_2_O_3_ calcined at 1073 K (direct heating).

According to the phase diagram, only the cubic phase was found in both oxides [11]. Figure 5 reveals that the gradual heating method caused peak broadening and had a major effect on the particle size, as can be seen in Figure 6. 

In gradual heating, the diffusion rates are smaller, and the tendency of forming smaller grains is higher. Those small grains grow as the temperature rises but not entirely homogeneously. On the other hand, in direct heating, the kinetics are faster due to higher diffusion rates, and larger grains are mostly formed and grow. The dependency on the preparation method of the samples was also reported previously [20,32].

**Figure 7:**
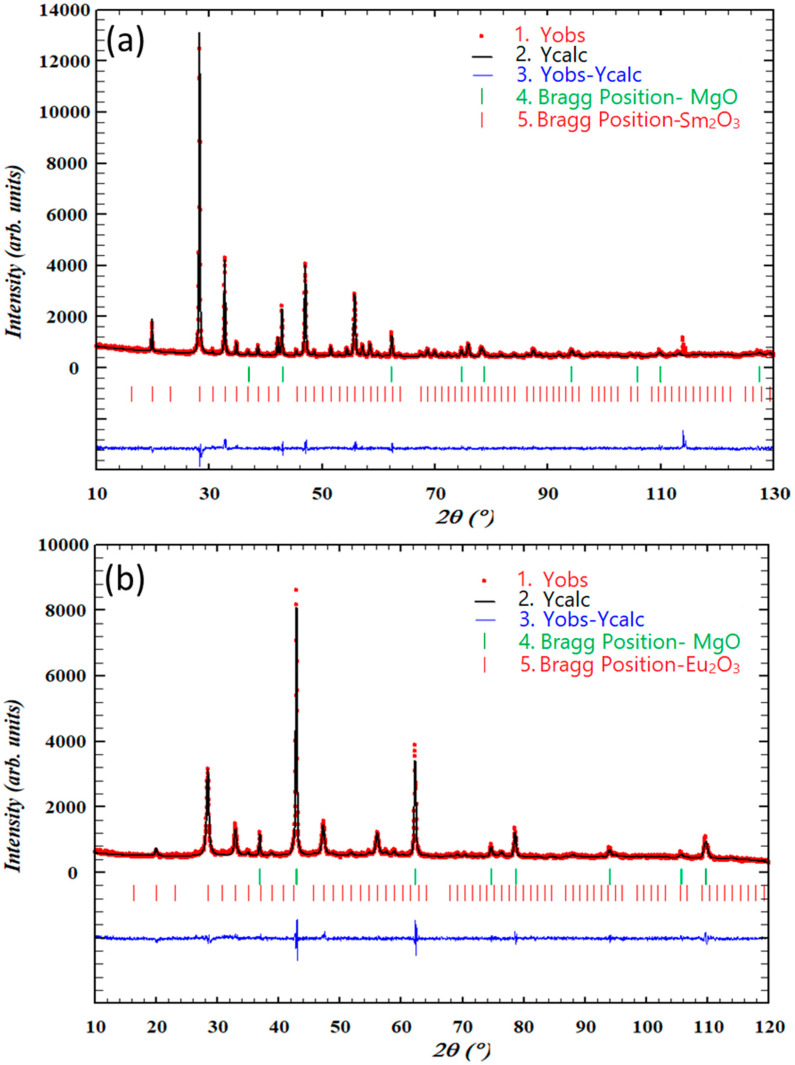
Rietveld refinements of (**a**) 40 vol % Sm_2_O_3_ in MgO calcined at 1273 K for 2 h and (**b**) 10 vol % Eu_2_O_3_ in MgO calcined at 1073 K for 2 h.

A Rietveld refinement was performed for each curve in Figure 1, Figure 2, Figure 3 and Figure 4. Representative cases of measured XRD pattern (Yobs), Rietveld refinement (Ycalc), and difference (Yobs-Ycalc) are shown in Figure 7.

**Figure 8:**
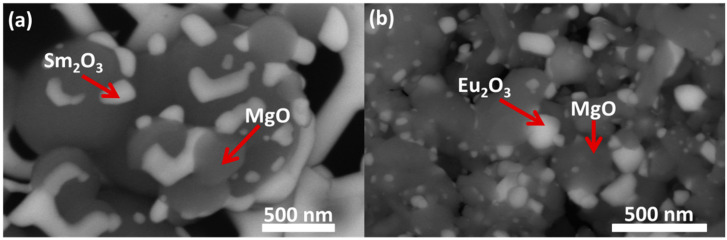
HR-SEM images in backscattered electrons (BSE) investigation mode of MgO with (**a**) 40 vol % Sm_2_O_3_ and (**b**) 40 vol % Eu_2_O_3_ calcined at 1473 K for 2 h.

HR-SEM images, presented in Figure 8, demonstrate the unique morphology of confided RE_2_O_3_ in a magnesia matrix. In all of the investigated samples, RE_2_O_3_ was mostly found as isolated grains embedded in the MgO matrix. A representative case, as shown in Figure 8, indicates that following 1473 K, nano-agglomerated particles with irregular shapes were observed. Energy dispersive X-ray spectroscopy (EDS) of these micrographs indicated the presence of nearly two separated pure phases: RE_2_O_3_ phase (brighter phase in Figure 8) and magnesia phase (darker phase in Figure 8), and a noteworthy phase boundary similar to previous reports [33]. These results are in agreement with the XRD results in Figure 1 and Figure 2, and indicate two distinct non-interacting phases of MgO and RE_2_O_3_.

## 4. Conclusions

This research investigated the crystallographic phase transformation of some rare-earth oxides in two different conditions: as embedded particles in MgO matrix and as pure oxides. XRD analysis allowed to demonstrate the transformation of crystallographic phase transitions and to approximate the relative amount of each phase as a function of concentration, temperature, and crystal size. From a practical perspective, it was possible to observe the differences between two situations (unhindered and hindered grain growth process) when embedding the desired phase in MgO in small amounts. It can be concluded that once the grain growth process is suppressed, the transition to monoclinic phase is delayed for the same calcination temperature compared with the pure oxide. Two methods to control the crystal growth process by changing the thermal treatment parameters were also investigated. Performing a direct heat treatment led to larger crystals, while a gradual heat treatment showed similar results to the RE_2_O_3_–MgO systems. In general, both suppression methods (embedding particles in MgO matrix or performing a gradual heat treatment process) ended in diverting the cubic-to-monoclinic transition towards higher temperatures. This indicates that grain size has a role in determining the crystallographic phase obtained. 

## Figures and Tables

**Table 1:** Inductively coupled plasma optic emission spectroscopy (ICP-OES) results.

RE_2_O_3_	Required vol % of RE_2_O_3_ in MgO	Theoretical wt% of RE_2_O_3_	ICP-OES results-wt% of RE_2_O_3_
Sm_2_O_3_	5	9.48	9.36 ± 0.74
	10	18.10	17.8 ± 0.9
	20	33.21	34.2 ± 1.7
	40	57.10	59.7 ± 3.0
Eu_2_O_3_	5	9.68	9.42 ± 0.47
	10	18.45	18.97 ± 0.95
	20	33.74	33.56 ± 1.68
	40	57.59	58.4 ± 2.92

**Table 2:** Analysis results (Sm_2_O_3_).

vol % of Sm_2_O_3_		Temperature (K)	d (nm)	Phase Composition (wt %)	Lattice Parameter (Å)
5	873		c-10.4	9.9% c + 90.1% MgO	a = b = c = 10.924
	1073		c-34.0	8.3% c + 91.7% MgO	a = b = c = 10.922
	1273		c-47.3	10.1% c + 89.9% MgO	a = b = c = 10.923
	1373		c-50.3 m-80.5	4.9% c + 6.0% m + 89.1% MgO	a = b = c = 10.920 a = 14.171 b = 3.625 c = 8.844 β = 100.04°
	1473		c-56.5 m-92.7	2.0% c + 9.0% m + 89.0% MgO	a = b = c = 10.911 a = 14.176 b = 3.624 c = 8.843 β = 100.04°
10	873		c-25.6	17.6% c + 82.4% MgO	a = b = c = 10.928
	1073		c-40.4	18.6% c + 81.4% MgO	a = b = c = 10.912
	1273		c-50.1	19.1% c + 80.9% MgO	a = b = c = 10.904
	1373		c-48.2 m-88.1	11.8% c + 5.8% m 82.4% MgO	a = b = c = 10.922 a = 14.168, b = 3.620, c = 8.844, β = 100.04°
	1473		c-64.7 m-104.5	1.3% c + 17.9% m 80.8% MgO	a = b = c = 10.918 a = 14.161, b = 3.626, c = 8.848, β = 100.04°
20	873		c-30.3	33.0% c + 67.0% MgO	a = b = c = 10.927
	1073		c-49.3	34.2% c + 65.8% MgO	a = b = c = 10.919
	1273		c-61.2	32.8% c + 67.2% MgO	a = b = c = 10.919
	1373		c-62.5 m-110.5	8.5% c + 25.2% m + 66.3% MgO	a = b = c = 10.924 a = 14.161, b = 3.620, c = 8.844, β = 100.04°
	1473		c-71.2 m-135.8	0.1% c + 33.1% m + 66.8% MgO	a = b = c = 10.922 a = 14.168, b = 3.624, c = 8.847, β = 100.05°
40	873		c-37.2	57.8% c + 42.2% MgO	a = b = c = 10.926
	1073		c- 56.3	58.1% c + 41.9% MgO	a = b = c = 10.921
	1273		c-70.2	56.5% c + 43.5% MgO	a = b = c = 10.917
	1373		c-63.4 m-120.7	20.4% c + 36.1% m + 43.5% MgO	a = b = c = 10.924 a = 14.159, b = 3.628 c = 8.841, β = 100.04°
	1473		m-144.5	56.8% m + 43.2% MgO	a = 14.165, b = 3.623, c = 8.845, β = 100.03°
pure Sm_2_O_3_	873		c-35.2	100% c	a = b = c = 10.930
	1073		c-53.4	100% c	a = b = c = 10.930
	1273		c-55.5 m-146.2	91.0% c + 9.0% m	a = b = c = 10.929 a = 14.176, b = 3.627, c = 8.852, β = 100.04°
	1473		m-177.3	100% m	a = 14.170, b = 3.626, c = 8.848, β = 100.04°

**Table 3:** Analysis results (Eu_2_O_3_).

vol % of Eu_2_O_3_	Temperature (K)	Approximated Average Crystal Size (nm)	Phase Composition (wt%)	Lattice Parameter (Å)
5	873	c-6.2	9.5% c + 90.5% MgO	a = b = c =10.867
	1073	c-10.8	9.9% c + 90.1% MgO	a = b = c 10.862
	1273	c-25.7	10.1% c +89.9% MgO	a = b = c 10.861
	1473	c-30.1	9.3% c + 90.7% MgO	a = b = c 10.861
10	873	c-18.4	17.7% c + 82.3% MgO	a = b = c 10.865
	1073	c-26.7	18.6% c + 81.4% MgO	a = b = c 10.863
	1273	c-36.4	18.2% c + 81.8% MgO	a = b = c 10.859
	1473	c-50.2	18.0% c + 82.0% MgO	a = b = c 10.859
20	873	c-21.1	32.8% c + 67.2% MgO	a = b = c 10.862
	1073	c-29.4	34.0% c + 66.0% MgO	a = b = c 10.861
	1273	c-49.9	33.5% c + 66.5% MgO	a = b = c 10.861
	1473	c-60.5	33.6% c + 66.4% MgO	a = b = c 10.857
40	873	c-33.4	57.8% c + 42.2% MgO	a = b = c 10.867
	1073	c-48.4	57.1% c + 42.9% MgO	a = b = c 10.856
	1273	c-66.4	58.6% c + 41.4% MgO	a = b = c 10.856
	1473	m-197, c-54.5	8.6% m + 49.5% c + 41.9% MgO	a = b = c 10.861 a = 14.108, b = 3.602, c = 8.808, β = 100.03°
pure Eu_2_O_3_	873	c-30.9	100% c	a = b = c 10.867
	1073	c-40.4	100% c	a = b = c 10.867
	1273	c-71.1	100% c	a = b = c 10.868
	1473	m-224.0, c-64.2	80.2% m + 19.8% c	a = b = c 10.867 a = 14.105, b = 3.601, c = 8.805, β = 100.04°
